# Association of Epoxide Hydrolase 2 Gene Arg287Gln with the Risk for Primary Hypertension in Chinese

**DOI:** 10.1155/2020/2351547

**Published:** 2020-02-28

**Authors:** Liang Ma, Hailing Zhao, Meijie Yu, Yumin Wen, Tingting Zhao, Meihua Yan, Qian Liu, Yongwei Jiang, Yongtong Cao, Ping Li, Wenquan Niu

**Affiliations:** ^1^Clinical Laboratory, China-Japan Friendship Hospital, Beijing, China; ^2^Beijing Key Laboratory of Immune-Mediated Inflammatory Diseases, Institute of Clinical Medical Sciences, China-Japan Friendship Hospital, Beijing, China; ^3^Department of Nephrology, China-Japan Friendship Hospital, Beijing, China; ^4^Department of Nephrology, Beijing Hepingli Hospital, Beijing, China; ^5^Institute of Clinical Medical Sciences, China-Japan Friendship Hospital, Beijing, China

## Abstract

**Background:**

Epoxide hydrolase 2 (*EPHX2*) gene coding for soluble epoxide hydrolase is a potential candidate in the pathogenesis of hypertension.

**Objectives:**

We aimed to assess the association of a missense mutation, R287Q, in *EPHX2*) gene coding for soluble epoxide hydrolase is a potential candidate in the pathogenesis of hypertension.

**Methods:**

This study involved 782 patients with primary hypertension and 458 healthy controls. Genotyping was done using TaqMan technique. Activity of soluble epoxide hydrolase fusion proteins was evaluated by the conversion of 11,12-EET to corresponding 11,12-DHET using ELISA kit.

**Results:**

After taking carriers of R287Q variant GG genotype as a reference, those with GA genotype had a significantly reduced risk of hypertension (adjusted odds ratio: 0.72, 95% confidence interval: 0.56 to 0.93, *P* = 0.013). Five significant risk factors were identified, including age, body mass index, total cholesterol, homocysteine, and R287Q variant. These five risk factors for hypertension were represented in a nomogram, with a descent prediction accuracy (C-index: 0.833, *P* = 0.013). Five significant risk factors were identified, including age, body mass index, total cholesterol, homocysteine, and R287Q variant. These five risk factors for hypertension were represented in a nomogram, with a descent prediction accuracy (C-index: 0.833,

**Conclusions:**

We provide evidence that R287Q mutation in *EPHX2* gene was associated with reduced risk of primary hypertension and low activity of soluble epoxide hydrolase.*EPHX2*) gene coding for soluble epoxide hydrolase is a potential candidate in the pathogenesis of hypertension.

## 1. Introduction

Hypertension is a polygenic multifactorial disease [1, 2]. Latest statistics indicate that global prevalence of hypertension has exceeded as high as 1.3 billion [3]. Early identification of at-risk individuals may allow for early interventions that might reduce the prevalence of hypertension and its associated complications [4, 5]. Considering the polygenic nature, it is of interest to identify and characterize genomic variants underlying predisposition to hypertension. Although numerous genome-wide association studies have been conducted to unravel the genetic basis of hypertension, the full picture is not yet clear [6–12]. A straightforward approach is to study likely functional variants in coding regions of hypertension-susceptibility genes.

Evidence is growing indicating that epoxide hydrolase 2 (*EPHX2*) gene coding for soluble epoxide hydrolase is a potential candidate in the pathogenesis of hypertension [13–15]. In humans, *EPHX2* gene encodes soluble epoxide hydrolase, which is a homodimer composed of two domains. The C-terminal domain imparts activity of soluble epoxide hydrolase, and the N-terminal domain is a phosphate domain [16]. There is evidence that the C-terminal hydrolase domain plays a significant role in blood pressure regulation, possibly via metabolizing lipids and other endogenousepoxide containing compounds [17, 18]. Animal studies showed that soluble epoxide hydrolase inhibitor, 12-(3-adamantan-1-y1-ureido)-do-decanoic acid (AUDA), was found to attenuate angiotensin II-induced hypertension [19], and *EPHX2* knockout mice exhibited lowered blood pressure and were immune from ventricular dysfunction [20]. It is hence reasonable to hypothesize that variation in *EPHX2* gene may be predictive of hypertension risk.

To test this hypothesis, we aimed to assess the association of a missense mutation at exon 8, R287Q (rs751141), in *EPHX2* gene with the risk of primary hypertension in Han Chinese and examine the association of this variant with enzyme activity of soluble epoxide hydrolase.

## 2. Materials and Methods

This is a hospital-based case-control association study. This study was approved by the institutional ethics committee of China-Japan Friendship Hospital.

### 2.1. Study Participants

This study involved a total of 1240 participants, who are of Han Chinese and were recruited from China-Japan Friendship Hospital between August 2016 and February 2018. All study participants were divided into the case group and the control group based on the presence and absence of primary hypertension, respectively. There were 782 patients with primary hypertension aged 63.03 years in the case group and 458 normotensive healthy participants aged 58.14 years in the control group. Each participant read and signed the informed consent form.

### 2.2. Diagnosis

Primary hypertension is defined as systolic blood pressure (SBP) measurement of ≥140 mmHg or diastolic blood pressure (DBP) ≥90 mmHg or self-reported usage of antihypertensive regimens. Normal blood pressure is defined as SBP measurement of <140 mmHg and DBP <90 mmHg. Blood pressure was measured at sitting position after a minimum rest of 10 minutes using a calibrated mercury sphygmomanometer with appropriate adult cuff size by certified examiners. Patients with any form of secondary hypertension based on the results of clinical laboratory and the diagnosis from physicians were excluded.

### 2.3. Data Collection

Anthropometric indexes including age, gender, ethnicity, body weight and height, SBP, and DBP were recorded or measured at the time of recruitment. Blood pressure was measured on three occasions, and the mean of the last two measurements was used for analysis. Body mass index (BMI) was calculated by dividing height (in meters) by weight (in kilograms) squared.

Serum concentrations of fasting total cholesterol (TC), triglycerides (TG), high-density lipoprotein cholesterol (HDL-C), low-density lipoprotein cholesterol (LDL-C), and homocysteine (Hcy) of each participant were assayed using an automated biochemical analyzer (AU5800 Clinical Chemistry System, Beckman Coulter, Brea, CA, USA) according to the manufacturer's instructions at the Clinical Laboratory of China-Japan Friendship Hospital.

### 2.4. DNA Extraction

Genomic DNA was extracted from peripheral blood samples using the QIAamp DNA Blood Mini Kit (Qiagen, Hilden, Germany) according to the manufacturer's instructions and then stored at −20°C or amplified immediately. The concentrations of genomic DNA were determined using the NanoDrop 1000 spectrophotometer (ThermoScientific, Waltham, MA, USA).

### 2.5. Genotyping

Genotypes of R287Q variant in *EPHX2* gene were determined using the TaqMan SNP Genotyping Assay (Applied Biosystems, Waltham, MA, USA). Specifically, 50 ng DNA was amplified in a 25 *μ*l reaction mixture containing 12.5 *μ*l of Premix Ex Taq (Probe qPCR) (Takara, Japan), 5 pmol of each primer (Applied Biosystems), and 3 pmol of each probe (Applied Biosystems) for the amplification of *EPHX2* gene. The primer and probe sequences were designed and synthesized by Applied Biosystems. The primer sequences were as follows: F: 5′-CGG GAG GAG CAG ATG ACT CT-3′; R: 5′-TGG AGT GTG CCT GTT TGT TTT C-3′. The probe sequences were as follows: FAM-5′-CAT AGC TAG GAC CCG GTA ACC TGC CT-3′-TAMRA and VIC-5′-CCA TAG CTA GGA CCT GGT AAC CTG CCT-3′-TAMRA. Amplification was performed using a real-time polymerase chain reaction (PCR) detector (LightCycler 480, Roche Diagnostics, Penzberg, Germany), with a PCR temperature profile consisting of denaturation at 95°C for 10 minutes following 40 cycles of denaturation at 95°C for 15 seconds and annealing and elongation at 65°C for 60 seconds.

To avoid genotyping misclassification, 50 DNA samples were randomly selected for direct sequencing. The genomic sequence containing the locus R287Q was amplified using the following primers: F:5′-TTA CAG GAA GAA GGG GAT GG-3′ and R:5′-GGC AGG TAG AAG GCA AGA CC-3′ according to standard protocols. PCR products were purified using the QIAquick PCR purification kit (Qiagen) and subsequently analyzed with an automated DNA sequencer (3500 Genetic Analyzer, Applied Biosystems).

### 2.6. Plasmid Construction

After PCR amplification and introduction of a 5′EcoRI restriction site and 3′ XhoIsite, wild-type human *EPHX2* was subcloned to the PUC-T vector, and R287Q variant *EPHX2* was subjected to site-directed mutagenesis using the QuickChange mutagenesis system. To generate human fusion proteins, wild-type and R287Q variant *EPHX2* cDNAs were subcloned into the EcoRI/XhoI sites of the pcDNA3.1/V5-His A vector. Plasmids were sequenced to verify proper insertion.

### 2.7. Enzyme Activity Assay

Activity of fusion proteins was evaluated by the conversion of 11,12-EET (EET: epoxyeicosatrienoic acid) to corresponding 11,12-DHET (DHET: dihydroxyeicosatrienoic acid) in HK-2 cells. HK-2 cells grown in six-well plates were transducted with soluble epoxide hydrolase fusion protein. Cells were exposed for 2 h to 1 *μ*M 11,12-EET, and culture medium was collected. 11,12-DHET was measured by using the 11,12-DHET immunoassay kit ELISA (Detroit R&D, Detroit, MI).

### 2.8. Statistical Analysis

Continuous data are expressed as mean (standard deviation) and compared using *t* test or Wilcoxon rank-sum (Mann–Whitney) test where appropriate. Categorical data are expressed as count (percentage) and compared using chi-squared test or Fisher exact test, which was also used to test the Hardy–Weinberg equilibrium and compare the genotype and allele distributions of R287Q variant in *EPHX2* gene. Association of this variant with the risk for hypertension was quantified using odds ratio (OR) with 95% confidence interval (CI) in logistic regression analysis before and after considering confounding factors. In addition, this association was also explored under additive, dominant, and recessive models of inheritance. Significant risk factors for hypertension were identified before and after using the propensity score matching method to account for some confounding factors using the forward logistic regression analysis. The prediction of risk factors for hypertension was visualized using restricted cubic spline method. To facilitate clinical assessment, a nomogram graph was generated on the basis of significant confounding factors [21].

## 3. Results

### 3.1. Baseline Characteristics

Table 1 shows the distributions of baseline characteristics between cases and controls. Distributions of age, BMI, SBP, and HCY were significantly higher in cases than in controls (*P* < 0.05). Distributions of gender composition, DBP, TG, HDLC, and LDLC were comparable between the two groups.

### 3.2. Distributions and Risk Prediction

The genotypes of R287Q variant in *EPHX2* gene were in the Hardy–Weinberg equilibrium, and the genetic distributions of this variant, as well as the risk prediction for hypertension, are presented in Table 2. Only marginal significance was noticed for the genotype and allele distributions between cases and controls (*P* = 0.032 and 0.023, respectively). After taking carriers of GG genotype of R287Q variant as a reference group, those with GA genotype had a significantly reduced risk of hypertension before and after adjusting for age, gender, and BMI (adjusted OR = 0.72, 95% CI: 0.56 to 0.93, *P* = 0.013).

Risk prediction of R287Q variant in *EPHX2* gene for hypertension was also examined under additive, dominant, and recessive models of inheritance (Table 3). Association of this variant with hypertension risk was statistically significant under additive and dominant models, even after adjusting for age, gender, and BMI. For instance, under the dominant model, this variant was associated with a 26% reduced risk for hypertension (adjusted OR = 0.74, 95% CI: 0.58 to 0.94, *P* = 0.014).

### 3.3. Significant Risk Factors

Forward logistic regression analysis was undertaken to identify potential risk factors for hypertension. Before propensity score matching, age, BMI, TC, HCY, and R287Q variant were associated with the significant risk of hypertension (Table 4). After propensity score matching on age, BMI, and TC (Supplementary [Supplementary-material supplementary-material-1]), significance retained for both HCY (OR = 1.28, 95% CI: 1.10 to 1.49, *P* = 0.001) and R287Q variant (OR = 0.70, 95% CI: 0.56 to 0.88, *P* = 0.002).

Additionally, after matching for age, BMI, and TC, the genotype and allele distributions of R287Q variant remained statistically significant between cases and controls, and the risk prediction was more obvious than that before matching especially under the dominant model of inheritance (OR = 0.65, 95% CI: 0.49 to 0.86, *P* = 0.002) (Supplementary [Supplementary-material supplementary-material-1]).

### 3.4. Nomogram Graph

**Figure 1:**
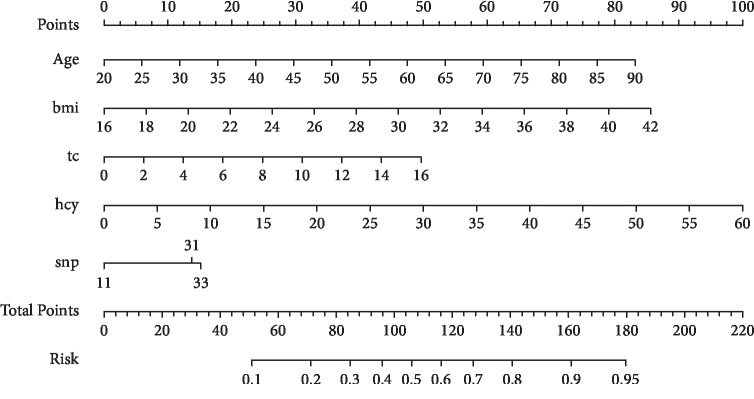
The nomogram graph of significant risk factors in association with hypertension.bmi, body mass index; tc, total cholesterol; hcy, homocysteine; snp, rs751141.

On the basis of five significant risk factors (age, BMI, TC, HCY, and R287Q variant), a nomogram graph was generated to quantify the association magnitude of individual factors and in combination with the risk for hypertension (Figure 1). The C-index for this nomogram graph was 0.833 (*P* < 0.001).

### 3.5. Activity Assay of Soluble Epoxide Hydrolase

**Figure 2:**
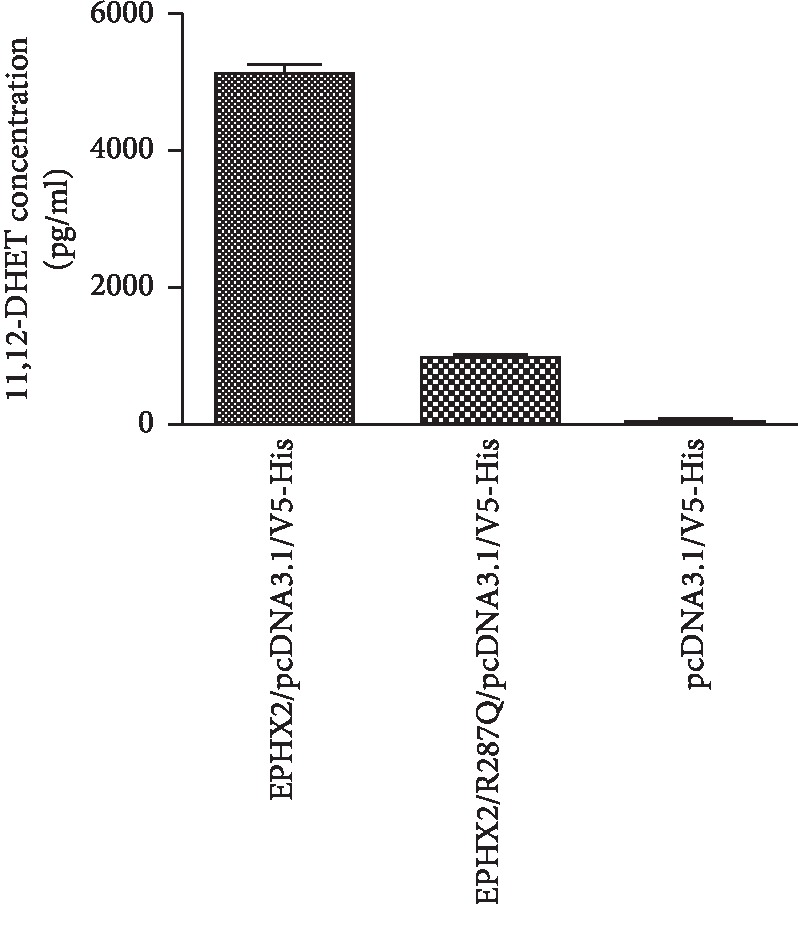
Enzyme activity assay of soluble epoxide hydrolase.

Enzyme activity of soluble epoxide hydrolase was evaluated in both wild-type group (EPHX2/pcDNA3.1/V5-His) and (R287Q variant) mutation group (EPHX2/R287Q/pcDNA3.1/V5-His). The ability of different soluble epoxide hydrolase fusion proteins to convert 11,12-EET to corresponding 11,12-DHET was measured. As shown in Figure 2, the 11,12-DHET levels were 5125 pg/ml (standard deviation: 136.1) in the wild type group, 955.1 pg/ml (standard deviation: 33.27) in the mutation group, and 51.04 pg/ml (standard deviation: 5.42) in the control group. The enzyme activity of soluble epoxide hydrolase was significantly lower in the mutation group than in the wild-type group (*P* < 0.001).

## 4. Discussion

The aim of this case-control study was to assess the association of R287Q variant in *EPHX2* gene with the protein activity of soluble epoxide hydrolase and the risk of hypertension. Our key findings indicate that the mutation of R287Q variant was associated with low enzyme activity of soluble epoxide hydrolase and reduced risk of having primary hypertension especially under the dominant model of inheritance, indicating that R287Q variant is a functional locus involved in the regulation of *EPHX2* gene expression.

Data on variants in *EPHX2* gene associated with human hypertension risk are very sparse in the current medical literature. Zhu and colleagues examined the association of *EPHX2* gene R287Q variant with the risk for primary hypertension in three ethnic groups (Uygur, Kazakh, and Han) from Xinjiang, and this variant was identified as an independent protective factor in Han Chinese only [22]. The present study confirmed the positive association between *EPHX2* gene R287Q mutation and reduced hypertension risk in a Han Chinese population, and extending previous findings, we noted that this association was more obvious under the dominant model of inheritance. Furthermore, we have examined the association of R287Q variant with the enzyme activity of soluble epoxide hydrolase and found a significant lower level of the 11,12-DHET in the mutation group than in the wild-type group. Our findings are biologically plausible, as soluble epoxide hydrolase is an enzyme responsible for rapid conversion of cytochrome P450 arachidonic acid epoxygenase metabolites, to inactive or less active DHETs [23, 24]. For practical reasons, our findings represent a novel approach to understanding molecular mechanisms involved in the pathogenesis of hypertension and may help target therapies according to the genotypes of R287Q variant for patients with primary hypertension to optimize prevention strategies. Based on above evidence, it is hence tempting to speculate that *EPHX2* gene R287Q variant, if involved, might contribute to the development of primary hypertension via affecting the enzyme activity of soluble epoxide hydrolase.

Considering the fact that hypertensive patients are older than healthy controls in this study, besides statistical adjustment, we employed propensity score matching technique to balance potential confounding factors between cases and controls in order to mimic a randomized controlled trial [25, 26]. Using this technique, the significant association between R287Q variant and hypertension risk retained, indicating the robustness of our findings. Besides R287Q variant, we additionally identified four risk factors in significant association with hypertension, and to facilitate clinical interpretation, we quantified and presented the contribution of five significant risk factors to the risk for primary hypertension using a nomogram graph, and this graph has a descent prediction accuracy. The adoption of this nomogram calculator for indicating the potential of having hypertension may enable practitioners to detect at-risk individuals and prompt aggressive prevention strategies [27] and further reduce the occurrence of the disease and greatly affect future health of these individuals.

The results of this study should be interpreted within the context of its potential limitations. Firstly, this study is cross-sectional in design, which precludes further comments on causality. Secondly, all study participants were enrolled from a single hospital, which might yield a possibility of population stratification. Thirdly, this study involves Chinese of Han ethnicity, and extrapolation to other ethnic groups is restricted. Fourthly, only an exonic variant in *EPHX2* gene was genotyped in this study, and it is of added interest to incorporate more variants in this gene to further interrogate haplotype-based contribution to hypertension risk.

Taken together, we provide genetic evidence that the mutation of R287Q variant in *EPHX2* gene was associated with low enzyme activity of soluble epoxide hydrolase and reduced risk of having primary hypertension. Further investigations in other ethnic or racial populations are needed to confirm the findings of this study and examine molecular mechanisms of *EPHX2* gene and hypertension.

## Figures and Tables

**Table 1:** Baseline characteristics of study participants.

Characteristics	Cases (*n* = 782)	Controls (*n* = 458)	*P*
Age (years)	63.03 (10.91)	58.14 (10.54)	<0.001
Male gender	467 (59.72%)	269 (58.73%)	0.733
BMI (kg/m^2^)	26.44 (3.50)	25.12 (3.51)	<0.001
SBP (mmHg)	137.71 (18.57)	127.97 (14.06)	<0.001
DBP (mmHg)	78.51 (10.99)	77.56 (8.79)	0.116
TC (mmol/L)	4.25 (1.28)	4.40 (1.20)	0.036
TG (mmol/L)	2.14 (1.33)	2.19 (1.47)	0.533
HDLC (mmol/L)	1.37 (1.11)	1.29 (1.03)	0.218
LDLC (mmol/L)	2.01 (1.01)	2.08 (1.00)	0.224
HCY (mmol/L)	13.81 (5.94)	11.86 (4.38)	<0.001

BMI, body mass index; SBP, systolic blood pressure; DBP, diastolic blood pressure; TC, total cholesterol; TG, triglyceride; HDLC, high-density lipoprotein cholesterol; LDLC, low-density lipoprotein cholesterol; HCY, homocysteine. Data are expressed as mean (standard deviation) for continuous variables and number (percentage) for categorical variables.

**Table 2:** The genotype and allele distributions of polymorphism rs751141 between cases and controls, as well as its risk prediction for hypertension risk before and after adjusting for confounding factors.

rs751141	Cases	Controls	*P*	OR (95% CI) *P*	Adjusted OR (95% CI) *P*^*∗*^
Genotype					
GG	472 (60.36%)	242 (52.84%)		Reference	Reference
GA	257 (32.86%)	182 (39.74%)	0.032	0.72 (0.57, 0.93) 0.010	0.72 (0.56, 0.93) 0.013
AA	53 (6.78%)	34 (7.42%)		0.80 (0.51, 1.26) 0.337	0.81 (0.50, 1.31) 0.395

Allele					
G	1201 (76.79%)	666 (72.71%)	0.023	Reference	
A	363 (23.21%)	250 (27.29%)		0.81 (0.67, 0.97) 0.023	

OR, odds ratio; 95% CI, 95% confidence interval. ^*∗*^*P* was adjusted for age, sex, and body mass index.

**Table 3:** Risk prediction of rs751141 polymorphism under different genetic models before and after adjusting for confounding factors.

rs751141	Before adjustment	After adjustment^*∗*^
Additive model	0.81 (0.68, 0.98) 0.027	0.82 (0.67, 0.99) 0.038
Dominant model	0.74 (0.58, 0.93) 0.010	0.74 (0.58, 0.94) 0.014
Recessive model	0.91 (0.58, 1.42) 0.667	0.92 (0.58, 1.47) 0.736

Data are expressed as odds ratio (95% confidence interval) *P*. ^*∗*^*P* was adjusted for age, sex, and body mass index.

**Table 4:** Identification of significant risk factors for hypertension before and after propensity score matching.

Risk factors	OR	95% CI	*P*
Age (per 5 years increment)	1.27	1.20–1.35	<0.001
Body mass index (per 3 kg/m^2^ increment)	1.48	1.32–1.66	<0.001
Total cholesterol (per 1 mmol/L increment)	1.14	1.00–1.29	0.045
Unmatched			
Homocysteine (per 5 mmol/L increment)	1.40	1.22–1.60	<0.001
rs751141	0.82	0.68–1.00	0.050
Matched^*∗*^			
Homocysteine (per 5 mmol/L increment)	1.28	1.10–1.49	0.001
rs751141	0.70	0.56–0.88	0.002

OR, odds ratio; 95% CI, 95% confidence interval; BMI, body mass index; TC, total cholesterol; HCY, homocysteine. ^*∗*^Matched variables: age, BMI, and TC.

## Data Availability

Data used to support the findings of this study will be available upon reasonable request.

## Supplementary Materials

Supplementary Materials Supplementary Figure 1: propensity score graph after matching for age, sex, body mass index, and total cholesterol. Supplementary Table 1: the genotype and allele distributions of polymorphism rs751141 between cases and controls, as well as its risk prediction for hypertension risk after using the propensity score matching method.
