# Supplementary material for: Association of Epoxide Hydrolase 2 Gene Arg287Gln with the Risk for Primary Hypertension in Chinese
Source: Int J Hypertens. 2020 Feb 28;2020:2351547. doi: 10.1155/2020/2351547 (PMC7064850; doi:10.1155/2020/2351547)
Supplement: Supplementary Materials — Supplementary Figure 1: propensity score graph after matching for age, sex, body mass index, and total cholesterol. Supplementary Table 1: the genotype and allele distributions of polymorphism rs751141 between cases and controls, as well as its risk prediction for hypertension risk after using the propensity score matching method. [file 2351547.f1.pdf]

Supplementary Figure 1. Propensity score graph after matching age, sex, body mass index and total cholesterol.

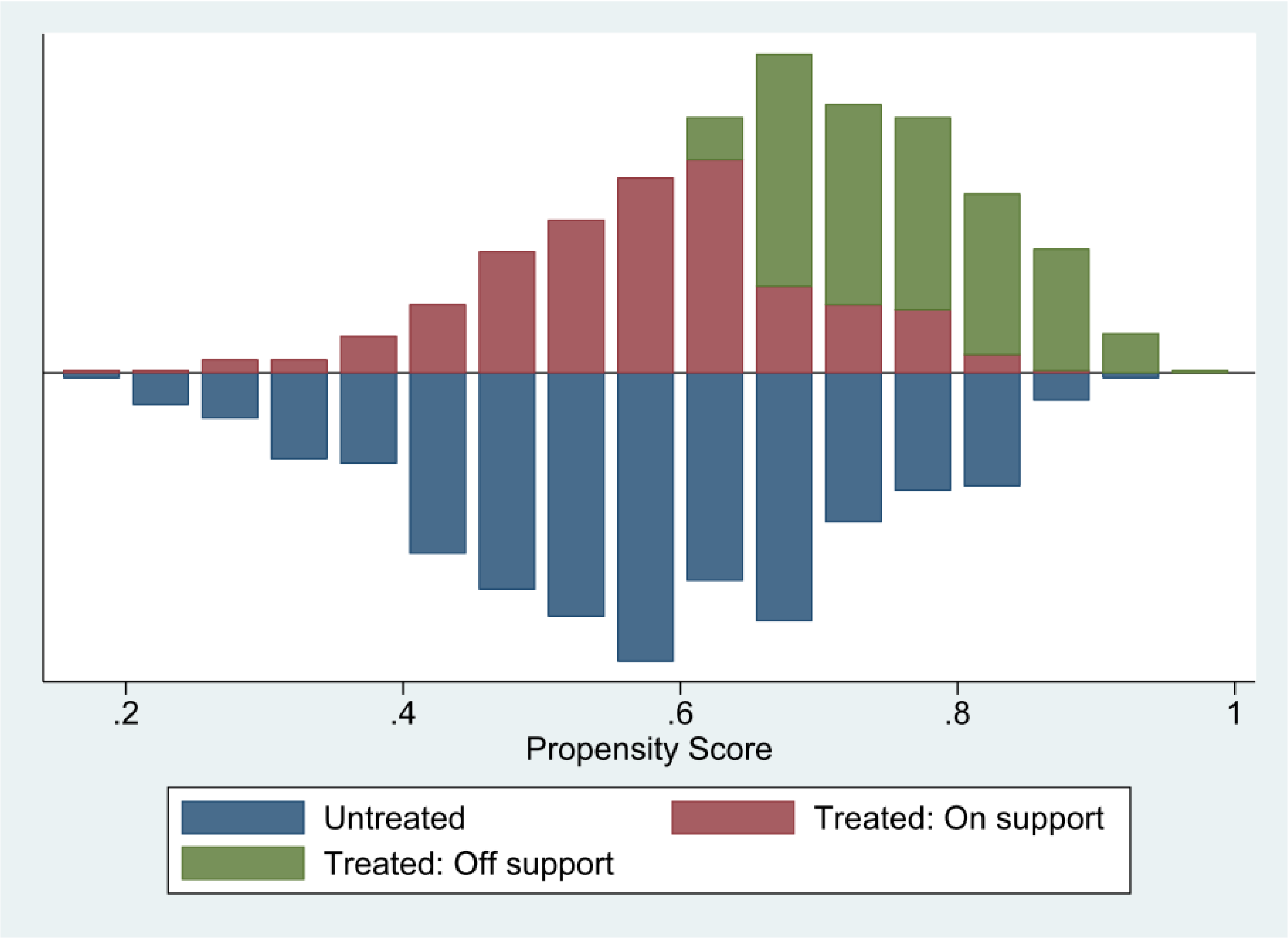

Supplementary Table 1. The genotype and allele distributions of polymorphism rs751141 between cases and controls, as well as its risk prediction for hypertension risk after using propensity score matching method.

**rs751141**	**Cases**	**Controls**	**P**	**OR (95% CI) P**	**Model**	**OR (95% CI) P**
Genotype						
GG	256 (63.68%)	213 (53.12%)		Reference	Additive	0.72 (0.57, 0.90) 0.004
GA	122 (30.35%)	155 (38.65%)	0.010	0.65 (0.49, 0.88) 0.006	Dominant	0.65 (0.49, 0.86) 0.002
AA	24 (5.97%)	33 (8.23%)		0.61 (0.34, 1.06) 0.077	Recessive	0.71 (0.41, 1.22) 0.214
Allele						
G	634 (78.86%)	581 (72.44%)	0.003	Reference		
A	170 (21.14%)	221 (27.56%)		0.70 (0.56, 0.89) 0.003		

Abbreviations: OR, odds ratio; 95% CI, 95% confidence interval.
